# The Prognostic Value of Hematological, Immune-Inflammatory, Metabolic, and Hormonal Biomarkers in the Treatment Response of Hospitalized Patients with Anorexia Nervosa

**DOI:** 10.3390/nu17142260

**Published:** 2025-07-09

**Authors:** Joanna Rog, Kaja Karakuła, Zuzanna Rząd, Karolina Niedziałek-Serafin, Dariusz Juchnowicz, Anna Rymuszka, Hanna Karakula-Juchnowicz

**Affiliations:** 1 I Department of Psychiatry, Psychotherapy and Early Intervention in Lublin, Medical University of Lublin, 20-059 Lublin, Poland; rog.joann@gmail.com (J.R.); kaja.karakula@umlub.pl (K.K.); karolaniedzialek@gmail.com (K.N.-S.); hanna.karakula-juchnowicz@umlub.pl (H.K.-J.); 2 Department of Psychiatry and Psychiatric Nursing, Medical University of Lublin, 20-059 Lublin, Poland; dariusz.juchnowicz@umlub.pl; 3 Department of Animal Physiology and Toxicology, Faculty of Medicine, The John Paul II Catholic University of Lublin, 20-950 Lublin, Poland; anrym@kul.pl

**Keywords:** eating disorders, inflammation, peripheral biomarkers, immune system, endocrine system, lipid metabolism, malnutrition

## Abstract

**Background/Objectives**: Anorexia nervosa (AN) is a chronic eating disorder with the highest mortality rate among psychiatric conditions. Malnutrition and starvation lead to long-term impairments in metabolic processes, hormonal regulation, and immune function, offering potential diagnostic and prognostic value. This study aimed to identify immune–metabolic–hormonal markers associated with treatment response and nutritional rehabilitation. **Methods**: Fifty hospitalized female patients with AN were included. Anthropometric measurements and venous blood samples were collected at admission and discharge, following partial nutritional recovery. Blood analyses included complete blood count, serum levels of total cholesterol, LDL and HDL, triglycerides, glucose, NT-pro-BNP, TSH, free thyroxine (fT4), sodium, chloride, potassium, calcium, iron, and vitamin D. Composite immune-inflammatory indices calculated were neutrophil-to-lymphocyte (NLR), monocyte-to-lymphocyte (MLR), platelet-to-lymphocyte (PLR); neutrophil-to-high-density lipoprotein (NHR), monocyte-to-high-density lipoprotein (MHR), platelet-to-high-density lipoprotein (PHR) and lymphocyte-to-high-density lipoprotein (LHR) ratios; systemic immune-inflammation (SII), and systemic inflammation response (SIRI) indexes. **Results**: Responders (R) and non-responders (NR) differed significantly at baseline in levels of sodium, chloride, fT4, monocyte count, MCV, NLR, MLR, SII, and SIRI (all: R < NR; *p* < 0.05). Predictive ability for treatment response was confirmed by AUC values (95%CI): sodium = 0.791 (0.622–0.960), chloride = 0.820 (0.690–0.950), fT4 = 0.781 (0.591–0.972), monocytes = 0.785 (0.643–0.927), MCV = 0.721 (0.549–0.892), NLR = 0.745 (0.578–0.913), MLR = 0.785 (0.643–0.927), SII = 0.736 (0.562–0.911), SIRI = 0.803 (0.671–0.935). The lower levels of inflammation and chloride are particularly predictive of better nutritional recovery, accounting for 26% of the variability in treatment response. **Conclusions**: The study demonstrated important insights into the hematological, metabolic, hormonal, and immune-inflammatory mechanisms associated with nutritional recovery in AN.

## 1. Introduction

Anorexia nervosa (AN) is a complex, multidimensional disorder characterized by severe and persistent abnormal eating behaviors, along with distressing thoughts and emotions related to food and body image [1]. As a chronic and relapsing condition, AN exhibits a lifetime prevalence ranging from 0.8% to 6.3% in females and from 0.1% to 0.3% in males [2]. The prevalence has notably increased following global crises as was the COVID-19 Pandemic [3]. AN has the highest mortality rate of psychiatric disorders, with suicide accounting for 13.9% of all deaths in patients with this eating disorder [4]. AN is associated with many physiological alterations, including abnormalities in immune function and inflammation [5].

Numerous factors contribute to the development and progression of AN; however, the clear etiology remains unknown. Malnutrition and starvation lead to disturbances in metabolic and endocrine parameters, resulting in long-term impairments in metabolic processes, hormonal regulation, and immune function. Biomarkers including, glucose, thyroid hormones, and electrolytes, can indicate systemic physiological dysregulation related to the severity of AN and serve as indicators for tracking the effectiveness of nutritional rehabilitation [6,7,8]. Changes in endocrine–metabolic pathways are linked to inflammation, which plays a significant pathophysiological role in AN and may contribute to the persistence of the disorder [9]. Peripheral inflammation biomarkers, such as blood count values (CBC), lipoproteins, and inflammatory ratios, have garnered attention as potential indicators of the inflammatory processes underlying AN [5]. They can offer potential for both diagnostic and prognostic utility in AN. The relationship between immune function, in endocrine–metabolic pathways, and AN remains unclear, highlighting the need for further clarification and research.

### 1.1. Hematological Indicators of Inflammation

#### 1.1.1. Complete Blood Count

Complete blood count (CBC) parameters include red blood cells (RBC), white blood cells (WBC), and platelets (PLT). Changes in WBC subpopulations (neutrophils, lymphocytes, monocytes, eosinophils, and basophils), can indicate ongoing inflammatory activity [10]. In patients with AN, alterations in CBC are commonly observed, especially anemia and leukopenia [11], which have been shown to correlate with lower body mass index (BMI) [12]. Leukopenia is more frequently observed in patients with the restricting subtype of AN [13]. Although disturbances in WBC count are observed, patients do not appear to have an increased susceptibility to infections [14]. Notably, most hematological and morphological abnormalities tend to resolve fully and promptly following adequate nutritional rehabilitation [12,14]. Monocytes (MON) seem to play a key role in the inflammatory response observed in AN. Monocytes have been associated with both the acute and chronic phases of inflammation. They produce inflammatory cytokines, particularly tumor necrosis factor-alpha (TNF-α), indicating an immune activation that exceeds what is typically observed in primary malnutrition. Alterations in MON gene expression profiles suggest a unique immunological contribution to the pathophysiology of AN [15].

#### 1.1.2. Neutrophil-to-Lymphocyte Ratio

The neutrophil-to-lymphocyte ratio (NLR) is calculated by dividing the absolute neutrophil count by the absolute lymphocyte count measured in peripheral blood. Reference values for this parameter vary across studies and populations. Some authors suggest reference NLR values range between 1.0 and 3.0, including pediatric populations [16]. Jaszczura et al. (2019) determined the optimal cut-off value for children to be 2.73 and showed a high specificity (90%) [17]. In 2017, the suggested optimal NLR range in healthy adults was established as 0.78 to 3.53. [18]. In some studies, reference ranges for the NLR have been shown to vary depending on sex and age, with males typically exhibiting higher values. NLR values exceeding 3.0 or below 0.7 may indicate an ongoing pathological immune-related process. Values within the range of 2.3 to 3.0 are often considered a ‘grey zone’ and may serve as an early warning sign, warranting further clinical evaluation [19]. Elevation in this index can be associated with low-grade chronic inflammation (LGI) and was connected to the prognosis of various conditions, including cardiovascular diseases [20], cancer [21], infection, atherosclerosis [22], trauma [23], postoperative complications [24], smoking [25] and psychiatric disorders [26]. Based on 8715 participants, the Rotterdam study concluded that NLR levels were independently and significantly associated with an increased risk of all-cause mortality [25].

Several studies have reported lower values of the NLR in AN patients compared to healthy controls [1,11]. Lower NLR values have been proposed to be a possible predictor of AN severity [1], and according to a pre-print manuscript, may serve as a biomarker for monitoring recovery [27]. However, the mean values of NLR in AN could be assigned to a normal range as identified by the previously mentioned authors. The results are not homogeneous throughout the studies. Khalil et al. (2017) observed elevated NLR values in individuals with AN and a history of childhood maltreatment and emotional abuse [28]. Additionally, a 2021 study observed that an increase in NLR was a significant and independent predictor of total body decreased bone mineral density in AN course [29].

#### 1.1.3. Monocyte-to-Lymphocyte Ratio

The monocyte-to-lymphocyte ratio (MLR) is calculated by dividing the absolute monocyte count by the absolute lymphocyte count in peripheral blood. There are currently no universally accepted numerical reference cut-off values of MLR. Values reported in the literature range from 0.2 to 0.4, with variability depending on the studied population and clinical context [30,31]. Most cut-off points were established based on the clinical population, which challenges the estimation of the predictive range for MLR. An elevated MLR may reflect LGI and has been proposed as a prognostic marker for mortality rate, and conditions such as heart failure [32], tuberculosis [30], lymphoma [33], and psychiatric disorders [34].

#### 1.1.4. Platelet-to-Lymphocyte Ratio

The ratio of the absolute platelet count to the absolute lymphocyte count (PLR) is influenced by age and sex. In healthy pregnant women, reference values typically range from 90 to 210 [35]. In the Chinese population, the observed range is approximately 36.63 to 149.13 for men and 43.36 to 172.68 for women [36]. Elevation of PLR can be used in the assessment of inflammatory activity, including autoimmune, atherosclerotic, metabolic, prothrombotic, neoplastic, and psychiatric diseases [37,38,39]. It was found to be a significant and independent predictor of decreased total body and lumbar bone mineral density in AN [29].

#### 1.1.5. Systemic Immune-Inflammation Index

The systemic immune-inflammation index (SII) is calculated by multiplying the platelet count by the neutrophil count and then dividing the result by the lymphocyte count in peripheral blood. The reference range and cut-off point are challenging to identify. A conclusion based on a study of 250 healthy adults indicates that the SII ranges from 253.7 to 373.9 [40]. In other studies, involving various patients, the cut-off value of the SII used for outcome prognosis ranges from 390 to 410 [41]. The SII reflects the balance between inflammation and immune response, indicating pro- and anti-inflammatory homeostasis. A high SII is associated with a poorer prognosis in various diseases, including cancer [42], cardiovascular [43] and psychiatric diseases [44].

#### 1.1.6. Systemic Inflammation Response Index

The systemic inflammation response index (SIRI) is calculated by multiplying the monocyte count by the neutrophil count and dividing the result by the lymphocyte count. The SIRI represents the equilibrium between systemic inflammatory activity and immune system regulation [45]. There is no universally established cut-off point for SIRI, as values may vary depending on the studied population and disease-related context. In clinical research across various patient groups, the cut-off values for prognostic assessment typically range up to 1.0 [46]. An elevated SIRI indicates a predominant inflammatory state, and has been observed in cancer [47], cardiovascular diseases [48], infections, and psychiatric disorders, including depression [49] and bipolar disorder [50].

### 1.2. Hematological-Lipid Indices

CBC- and lipid-based composite indices refer to a set of hematological and biochemical markers that combine parameters from both the immune and lipid profiles. The biomarkers reflect the interplay between inflammation, immune response, and lipid metabolism, particularly under pathological conditions such as psychiatric disorders (e.g., eating disorders), cardiovascular disease, and cancer. Commonly investigated include:

#### 1.2.1. Neutrophil-to-High-Density Lipoprotein Cholesterol Ratio

Neutrophil-to-high-density lipoprotein cholesterol ratio (NHR) is calculated by dividing the absolute neutrophil count by the concentration of high-density lipoprotein cholesterol (HDL-C). This composite marker reflects inflammation and lipid metabolism [51], which play key roles in the pathogenesis of chronic LGI-related disorders, including cardiometabolic, disorders. Although no established reference range for NHR exists, several studies have proposed diagnostic and prognostic cut-off values. Thresholds associated with coronary artery disease have been reported at 1.51 [52] and 3.87 [53], while higher values such as 5.74 [54] and 11.28 [55] have been suggested for predicting adverse cardiac events. In 2024, cut-off points for metabolic syndrome were determined at 1.29 for men and 1.13 for women [56]. In 2024, researchers determined the cut-off point for metabolic syndrome at 1.29 for men and 1.13 for women [56]. NHR is a strong predictor of poor cardiovascular outcomes [57,58,59] and cardiovascular mortality, atherosclerosis [57], cancers [58], diabetes, neurodegenerative diseases, and metabolic syndrome [56].

#### 1.2.2. Monocyte-to-High-Density Lipoprotein Cholesterol Ratio

The monocyte-to-high-density lipoprotein cholesterol ratio (MHR) is a biomarker to assess systemic inflammation and oxidative stress [59]. To calculate MHR, the absolute number of monocytes needs to be divided by the concentration of HDL-C. Although no standardized reference range has been established, values below 0.3 [60] are generally considered optimal in minimizing inflammation-related health risks. Elevated MHR values may serve as a predictor of poor prognosis for atherosclerosis and other inflammatory conditions, cardiovascular disease, renal disease, and dyslipidemia [61]. Higher MHR has also been linked to schizophrenia [62], major depressive disorder [63], and bipolar disorder [64].

#### 1.2.3. Platelet-to-High-Density Lipoprotein Cholesterol Ratio

The platelet-to-high-density lipoprotein cholesterol ratio (PHR) is calculated by dividing the absolute platelet count by the serum HDL-C concentration. It is a significant indicator of inflammation and a hypercoagulable state [65]. Biomarker has been positively associated with type 2 diabetes, prediabetes [66], heart failure [67], other cardiovascular diseases, non-alcoholic fatty liver disease, metabolic syndrome [65], and psychiatric disorders [68]. Although no universally established reference range for PHR exists, a 2024 study reported that values exceeding 111.49 were positively associated with cognitive impairment [69].

#### 1.2.4. Lymphocyte-to-High-Density Lipoprotein Cholesterol Ratio

The lymphocyte-to-high-density lipoprotein cholesterol ratio (LHR) is derived by dividing the absolute lymphocyte count by HDL-C concentration. Similar to PHR, there are no norms regarding the reference range for LHR. LHR may serve as a more reliable indicator of inflammatory burden and immunological competence than individual parameters alone [70]. A higher value of LHR has been associated with metabolic syndrome, cardiovascular risk [71], poor prognosis in sepsis [72], and psychiatric disorders [70].

Despite the growing body of evidence on low-grade inflammation (LGI) biomarkers derived from CBC and lipid profiles, studies assessing their relevance in eating disorders remain limited. Based on the exploratory objectives of this study, we hypothesize that specific hematological and inflammatory biomarkers are associated with treatment response and nutritional rehabilitation outcomes in female patients hospitalized with AN. These hypothesis-generating findings are intended to guide future adequately powered prospective studies.

The analysis included four main categories of peripheral blood biomarkers:

1. Hematological parameters, such as complete blood count indices including white blood cells (with differentials), red blood cells, hemoglobin, hematocrit, mean corpuscular volume (MCV), and platelet count.
2. Immune-inflammatory indices, calculated composite markers such as NLR, MLR, PLR, SII, SIRI, NHR, MHR, PHR, and LHR.
3. Metabolic parameters, including glucose, vitamin D, and electrolytes (sodium, chloride, potassium, calcium, and iron).
4. Hormonal parameters, specifically thyroid-stimulating hormone (TSH) and free triiodothyronine (fT3) and thyroxine (fT4).

By examining these markers, we seek to gain a deeper understanding of the inflammatory processes involved in AN, which could potentially offer insights into novel diagnostic or therapeutic approaches for this challenging disorder.

## 2. Materials and Methods

### 2.1. Participants

This prospective study was conducted between October 2022 and April 2024 at the I Department of Psychiatry, Psychotherapy and Early Intervention of the Medical University in Lublin, Poland. The medical staff is a multidisciplinary team that includes psychiatrists with both academic and clinical backgrounds, psychologists, psychotherapists, one dietitian, psychiatry residents, and psychiatric nurses. Patients are hospitalized either in the Adolescent Ward or the Adult Ward, depending on their age group. A total of 50 female patients aged 12 to 30 years were enrolled. All participants were hospitalized due to Anorexia Nervosa, diagnosed by a psychiatrist according to the ICD-10 criteria following a structured clinical interview. Written informed consent was obtained from all participants and, in the case of minors, from their legal guardians. Prior to study enrollment, each participant was informed about all study procedures and their voluntary participation.

Inclusion criteria were as follows:

- provision of informed consent by the participant or their legal guardian;
- female inpatient;
- age between 12 and 30 years;
- diagnosis of Anorexia Nervosa (F50) confirmed by a psychiatrist;

Exclusion criteria were as follows:

- diagnosis of an eating disorder other than AN that prevented clear classification.
- lack of informed consent.
- coexisting somatic diseases significantly affecting immune-inflammatory function (such as autoimmune disorders, malignancies, or acute infections), endocrine disorders in a decompensated state and other somatic conditions affecting electrolyte balance;
- pharmacological treatment known to alter hematological or lipid parameters (such as corticosteroids or immunosuppressants) within one month prior to hospitalization;
- diagnosed severe psychiatric disorders (e.g., psychosis, acute manic episodes, severe depression with suicidality), neurological diseases (e.g., epilepsy, neurodegenerative disorders, history of traumatic brain injury), or substance use disorders (including alcohol or illicit drugs).

All patients received care in accordance with a standardized clinical treatment program for AN implemented at the hospital, which included nutritional rehabilitation, medical monitoring, and psychotherapeutic support.

Anthropometric measurements (body weight and height) and venous blood samples were obtained within the first 1–3 days following admission (V0) and repeated 1–3 days before discharge (V1), after partial nutritional recovery. Due to variations in data completeness, the final sample size varied by parameter. For example, BMI was available for 49 participants at admission and for 44 participants at discharge. All procedures were approved by the Bioethics Committee of the Medical University of Lublin (ID: KE-0254/24/01/2022, KE-0254/58/02/2023) and conducted in accordance with the Declaration of Helsinki.

### 2.2. Biochemical Analysis

Peripheral venous blood samples were collected from fasting participants (after an 8 h overnight fast) at both time points (V0 and V1). All samples were processed in a single certified hospital laboratory and included

- Complete blood count (CBC);
- Metabolic parameters: serum levels of total cholesterol, lipoproteins (low-density and high-density), triglycerides, glucose;
- Endocrine and cardiovascular markers: TSH, fT4, N-Terminal pro b-type natriuretic peptide (NT-proBP);
- Nutritional status indicators: electrolytes (sodium, chloride, potassium), calcium, iron, and vitamin D concentration.

The following composite immune-inflammatory indices were calculated: NLR, MLR, PLR, SII, SIRI, NHR, MHR, PHR and LHR.

### 2.3. Statistical Analysis

Statistical analysis was conducted using the STATISTICA software package, version 13 (StatSoft Inc., Tulsa, OK, USA). The characteristics of the examined group were reported as mean ± standard deviation (SD), and as minimum and maximum (min–max) values for continuous variables, and as the number of participants (percentage, %) for categorical variables. Correlations between continuous variables were examined using the Pearson correlation coefficient (PCC). Due to unequal group sizes, differences in continuous variables between the two groups were assessed using the Mann–Whitney U test [73]. The receiver operating characteristic (ROC) curve was used to evaluate the ability of blood parameters to predict improved treatment response, defined as an increase in BMI, and was quantified by the area under the curve (AUC). The Youden index (sensitivity + specificity − 1) was used to determine the optimal cut-off points from the ROC analysis. To determine how potential predictors of response to nutritional rehabilitation influence each other and how they jointly contribute to the response, defined as changes in BMI, a stepwise multiple regression analysis was conducted. This approach also allowed for the assessment of how much of the variability in response could be explained by these predictors, taking their interrelations into account. The missing data were removed by listwise deletion. Statistical significance was defined as a two-sided test with *p* < 0.05.

## 3. Results

### 3.1. Clinical Characteristics of Participants

The characteristics of the examined group are presented in Table 1. The mean BMI before recovery was 15.12 ± 1.08 kg/m^2^. After treatment, the mean BMI increased to 17.36 ± 0.93 kg/m^2^. Thirty-seven individuals (74%) had the restricting subtype of AN, and twelve (24%) had the binge-eating/purging subtype. The duration of illness varied considerably among participants. Forty-three patients were taking medication, mainly selective serotonin reuptake inhibitors (SSRIs) (*n* = 30, 60%), antipsychotics (*n* = 17, 34%), tricyclic antidepressants (*n* = 15, 30%), antihistamines (*n* = 7, 14%), selective norepinephrine and serotonin reuptake inhibitors (SNRIs) (*n* = 3, 7%), mood stabilizers (*n* = 2, 4%), and other medications (*n* = 5, 10%). Thirty-seven individuals were taking at least one antidepressant drug.

### 3.2. Relationship Between Nutritional Status, Blood Parameters, and Sociodemographic Data

Age was positively correlated with the duration of hospitalization (r = 0.33; *p* < 0.05), and showed significant associations with several baseline (V0) blood parameters: negatively with white blood cell count (WBC; r = −0.82; *p* < 0.05) and lymphocyte count (r = −0.99; *p* < 0.05), and positively with total cholesterol (r = 0.86; *p* < 0.05), LDL cholesterol (r = 0.82; *p* < 0.05), monocyte-to-lymphocyte ratio (MLR; r = 0.94; *p* < 0.05), and lymphocyte-to-HDL ratio (LHR; r = −0.91; *p* < 0.05).

The duration of the hospitalization was strongly related to blood parameters at V1: chloride levels (r = −0.84; *p* < 0.05), mean corpuscular hemoglobin concentration (MCHC, r = 0.86, *p* < 0.05), platelet count (r = −0.91; *p* < 0.05), HDL cholesterol (r = 0.85; *p* < 0.05), and indexes: PLR (r = −0.91; *p* < 0.05), PHR (r = −0.94; *p* < 0.05).

BMI at V0 was inversely related to the duration of illness (r = −0.36; *p* < 0.05), as well as to fT4 at V0 (r = 0.84; *p* < 0.05), and calcium (r = −0.98; *p* < 0.05), TSH (r = 0.89; *p* < 0.05) at V1. BMI at V1 was inversely associated with iron levels at V0 (r = −0.90; *p* < 0.05).

Changes in BMI were related to SII (r = −0.94; *p* < 0.05), SIRI (r = −0.94; *p* < 0.05) and NHR (r = −0.95; *p* < 0.05) at V0, and to NT-proBNP (r = −0.95; *p* < 0.05), WBC (r = 0.89; *p* < 0.05) at V1.

At baseline, individuals receiving antidepressant medication exhibited higher MCHC and lower MCV compared to those not treated with antidepressants. Following nutritional rehabilitation, individuals undergoing antidepressant treatment demonstrated lower levels of fT3 and RBC as well as higher MCH and MCV, in comparison to their non-medicated counterparts (*p* < 0.05).

### 3.3. Potential Blood Indicators of Response to Nutritional Rehabilitation

Thirty-four individuals were classified into the responder group (R) (defined as a change in BMI of ≥1.5 kg/m^2^), and ten were assigned to the non-responder (NR) group. The 1.5 kg/m^2^ threshold was chosen as a clinically meaningful indicator of response. The responder and non-responder groups differed in baseline electrolytes concentration: sodium (R: median, M = 140.00 mmol/L; NR: M = 142.00 mmol/L; *p* = 0.005), chlorides (R: M = 102.30 mmol/L; NR: M = 105.05 mmol/L; *p* = 0.002), and fT4 concentration (R: M = 12.92 ng/L; NR: M = 16.25 ng/L; *p* = 0.009). The differences in CBC were also found: monocytes count (R: M = 0.38; NR: M = 0.41; *p* = 0.038), MCV (R: M = 86.80 fl; NR: M = 89.50 fl; *p* = 0.036) and inflammatory state: NLR (R: M = 0.94; NR: M = 1.54; *p* = 0.019), MLR (R: M = 0.17; NR: M = 0.26; *p* = 0.005), SII (R: M = 225.18; NR: M = 353.62; *p* = 0.024) and SIRI (R: M = 0.29; NR: M = 0.76; *p* = 0.003). After recovery, no significant differences in CBC parameters were observed between the groups, although the difference in inflammatory state indicators remained: MLR (R: M = 0.24; NR: M = 0.31; *p* = 0.036) and SIRI (R: M = 0.56; NR: M = 1.04; *p* = 0.048).

The ability of baseline concentration of sodium, chlorides, fT4, monocytes count, MCV and indexes: NLR, MLR, SII, and SIRI to predict treatment response in individuals with AN was evaluated using receiver operating characteristic (ROC) curve analysis, as shown in Table 2. The area under the curve (AUC) values with 95% confidence intervals were as follows: sodium = 0.791 (0.622–0.96); chlorides = 0.82 (0.69–0.95); fT4 = 0.781 (0.591–0.972); monocytes count = 0.785 (0.643–0.927); MCV = 0.721 (0.549–0.892); NLR = 0.745 (0.578–0.913); MLR = 0.785 (0.643–0.927); SII = 0.736 (0.562–0.911); SIRI = 0.803 (0.671–0.935). The proposed cut-off values for the most promising indicators of nutritional rehabilitation response are presented in Table 3 and Figure S1 (see supplementary data). The Youden indices of these blood parameters indicate moderate to good discriminatory ability.

### 3.4. The Effect of Blood Indicators of Response to Treatment on BMI Changes

To determine the complex interplay of potential indicators and confounders underlying changes in BMI, a multiple stepwise regression with forward elimination of independent variables from the full predictor model was performed. The following variables were included in the regression model: age, type of AN, duration of illness and hospitalization, sodium, chloride, fT4, monocyte count, MCV, NLR, MLR, SII, and SIRI. Changes in BMI from V0 to V1 were considered as the dependent variable. The results of the analysis are presented in Table 4. Among the variables, SIRI (*p* = 0.017) and chloride levels (*p* = 0.005) showed the strongest associations with BMI change. No significant effects were found for age, AN subtype, or the remaining blood parameters. Together, SIRI and chloride explained 26.41% of the variance in nutritional status improvement during hospitalization.

## 4. Discussion

Despite the rising knowledge and solid evidence supporting the role of immune-inflammatory processes, metabolic–endocrine disruptions, and lipid-related factors in the pathogenesis and prognosis of numerous chronic diseases, their clinical implications in AN remain understudied. The potential mechanisms of immune and metabolic dysregulation observed in individuals with eating disorders include alterations in the intestinal microbiota due to inadequate nutrition, dysregulation of the hypothalamic–pituitary–adrenal (HPA) axis related to chronic stress, and changes in cytokine levels and cell-mediated immunity caused by prolonged malnutrition [15]. The aim of this exploratory, hypothesis-generating study was to investigate potential prognostic inflammatory and metabolic–endocrine markers associated with treatment response in AN.

Our study included participants during the developmental stage and physical maturation. We examined not only the adolescent population, taking into account that physical development continues until approximately the age of 30. Neurodevelopmental processes, including the restructuring of neural pathways, are not complete until around the age of 25 [74]. Although the main maturation of the reproductive system typically ends between ages 16 and 20, HPA axis stabilizes closer to age 30 (25–30) [75]. Fluctuations in cortisol (the end product of HPA axis stimulation) and other hormones are thought to normalize approximately between 20 and 30 years of age. This time period is also critical for the differentiation of T and B lymphocytes, with full functional maturity of the immune system usually achieved by around 30 years of age [76]. Disruptions during this physiological developmental period may lead to irreversible changes throughout the body. Biological and neurohormonal immaturity may exacerbate clinical symptoms and reduce the effectiveness of therapeutic interventions in AN. Persistent and unrepaired brain disruption could negatively affect recovery and hinder the therapeutic process [77,78].

According to the results of our research, potential blood biomarkers that could predict improvement in nutritional status include electrolytes (chloride, sodium), CBC parameters (monocyte count and MCV), fT4, and inflammatory indexes, such as NLR, MLR, SII, and SIRI. Multiple regression analysis revealed that baseline chloride levels and inflammation (measured by SIRI) accounted for 26% of the variance in nutritional status improvement. The small sample size, particularly of the non-responder group (*n* = 10), limits the statistical power of the analysis and affects the stability of ROC AUC estimates. Our study provides an exploratory insight into the potential predictive value of selected blood parameters, rather than testablish definitive diagnostic cut-offs. The findings highlight potential markers which will be helpful to assess in further studies, as this study serves as an initial step to evaluate the feasibility and relevance of investigating these parameters in larger, adequately powered research.

The pro-/anti-inflammatory imbalance observed in the course of AN is supported by meta-analyses showing elevated levels of pro-inflammatory cytokines in patients with AN compared to healthy individuals [79]. These findings contrast with the well-established relationship between higher BMI, body fat mass, and increased inflammation [80]. The disruption of the pro-/anti-inflammatory state may partially contribute to clinically significant dysregulation of appetite, leading to appetite suppression in patients with AN. Nutrient intake affects T-cell metabolism and survival. Alterations in T-cell function and number lead to changes in adipokine levels, particularly leptin [81]. Adipokines, cytokines, and neuropeptides regulated by T-cells are known to mediate hunger and satiety [80]. Higher NLR ratio has been associated with greater body weight loss during the progression of the neoplastic process and has been suggested as a potential factor leading to worse outcomes and responses [82]. However, there is still a lack of studies examining the relationship between hunger-satiety regulation, neuropeptides, and inflammation in the course of AN.

The potential inflammation-related paradox is observed in other malnutrition-related states, where an increased ratio of cells expressing pro-inflammatory to regulatory cytokines has been reported. During childhood, malnutrition tends to be associated with higher concentrations of anti-inflammatory cytokines and less consistently altered levels of pro-inflammatory cytokines in blood, contrasting with the predominantly pro-inflammatory cytokine expression in the gut of malnourished children [83]. Elevated levels of inflammatory markers also diminish the positive effects of nutritional therapy and are proposed as markers of increased mortality linked with malnutrition [84]. This could have significant consequences in AN. The introduction of immune-regulatory compounds to the diet (such as nucleotides or vitamins) should be considered and evaluated in further studies.

Some of the markers in our study, which are potentially related to inflammation in various diseases, showed no predictive value in the examined population of patients with AN. The mechanisms linking LGI and AN could differ from those in typical metabolic disruptions, especially considering the undernutrition state of patients. Changes in hormonal, metabolic, and neurobiological processes during eating disorders can significantly affect the immune response. Although immunosuppression is suggested in AN, LGI could result from metabolic and psychological stress affecting the HPA axis, as well as tissue damage leading to the secretion of damage-associated molecular patterns (DAMPs) [28,85,86].

Inflammation is also associated with mood disorders and more severe depressive symptoms both in healthy individuals and patients with chronic diseases [87]. A lowered mood may contribute to greater resistance to therapy, a negative attitude toward treatment, and decreased motivation, which can ultimately lead to ineffective nutritional rehabilitation [88]. The inflammatory profile of patients with eating disorders is heterogeneous and varies across specific diagnosis subtypes [89]. Reducing the LGI state in patients with AN could potentially lower the risk of long-term consequences exacerbated by chronic inflammation, such as impaired glucose metabolism, loss of bone mineral density, and cognitive decline [69]. Inflammation-targeted interventions might help improve mood symptoms and appetite in patients with AN, potentially leading to a better response to nutritional rehabilitation as well [90].

Patients who responded better to treatment were characterized by significantly lower values of unspecific inflammatory markers (SIRI, NLR, MLR), both before and after nutritional rehabilitation. This may indicate that a less pronounced inflammatory response is associated with a more effective nutritional recovery. Our findings support the hypothesis that immune dysregulation and LGI play a role in the pathophysiology of AN [5]. Specifically, lower levels of inflammatory markers such as SIRI, NLR, and MLR observed in patients who responded better to treatment (the so-called “responders”) may reflect a less intense or “suppressed” inflammatory response in this subgroup [11].

The term “suppressed inflammatory response” in this context refers to a situation in which, despite the presence of physiological stressors that would typically activate the immune system (e.g., severe malnutrition, metabolic stress, hormonal disturbances), the body fails to mount a measurable inflammatory reaction detectable via classical peripheral blood markers. This blunted response may stem from overall immune exhaustion, cytopenias, adaptive immunological downregulation, or compensatory mechanisms that develop during chronic starvation [91].

This phenomenon presents a pathophysiological paradox that warrants further investigation. AN is characterized by systemic physical and psychological stress, which should, in theory, trigger immune activation and elevated inflammatory markers [92,93]. However, as shown in our study and others, a subset of patients—particularly those who respond better to nutritional rehabilitation—demonstrate unexpectedly low inflammatory index values. Long-term LGI may interfere with therapeutic efficacy and hinder treatment response.

This raises the possibility that some individuals may exhibit a more favorable “inflammatory profile,” marked by reduced or balanced immune activation, which could facilitate metabolic adaptation and improve treatment responsiveness [94]. Recognizing these subtypes may hold clinical significance for tailoring treatment strategies in the future.

Other assessed markers, such as fT4, MCV, monocyte count, and sodium levels, also showed potential as predictors of treatment response and are attractive for use in clinical settings due to their routine availability. Composite hematological-lipid indices (NHR, MHR, PHR, LHR) demonstrated variability but had limited predictive value in this study. Nonetheless, they may be useful in future research on long-term metabolic consequences of AN.

## 5. Strengths and Limitations of the Study

A major strength of this study is the comprehensive assessment of blood parameters in the examined group, which included not only immune-inflammatory biomarkers but also hormonal, lipid, metabolic, and nutritional indicators. This multidimensional evaluation allowed for a more integrative and in-depth analysis of the data. Fairly homogeneous group of patients and assessment in hospital conditions allow to minimize the impact of potential confounders.

Some limitations of the study should be considered when interpreting the results. The use of the term “recovery” in our paper refers to partial nutritional improvement, as assessed by changes in BMI. We acknowledge that this definition does not encompass psychological or behavioral recovery, and the absence of standardized psychometric assessments (i.e., validated eating disorder psychopathology scales) is a limitation that should be addressed in future research.

The sample size was relatively small (*n* = 50), which reduces the statistical power and limits the generalizability of the findings. The obtained results are hypothesis-generating and intended to inform the design of a future, adequately powered prospective study. Our study included only patients during their hospitalization, which likely increased their compliance with the nutritional intervention and could have influenced adherence to the guidelines we implemented. This specific setting and patient selection may have introduced selection bias and should be taken into account when interpreting the results. The AN more frequently affects women. Therefore, we focused our study exclusively on this gender, as extrapolating these results to the male population may be unreliable. The absence of a healthy control group makes it more difficult to determine the specificity of the observed associations. Furthermore, the study population was limited to female patients, preventing conclusions from being extended to males. The data were collected from a single clinical center, which may limit the environmental and demographic variability of the sample. The range of laboratory tests was restricted to routine parameters, and therefore key markers such as cytokines or indicators of hypothalamic–pituitary–adrenal axis activity (e.g., cortisol) were not included [95,96]. Real-time analysis was performed due to our intention to further use it in clinical practice, where results are always obtained in real-time. Nevertheless, batch effects were occurring. To ensure reliability and accuracy, validation of the results should be conducted by processing samples under exactly the same laboratory conditions with a blinded sample approach or by applying additional statistical correction methods. Moreover, the variability in the timing of blood sampling relative to admission or discharge (ranging from 1 to 3 days) may have affected the results. Likewise, concurrent use of medication by participants represents a potential confounding factor. Future studies could also include clinical symptoms of eating disorders and more precise assessments of nutritional status, such as bioelectrical impedance analysis.

## 6. Conclusions

The findings of our study indicate that immune-inflammatory, metabolic, and hormonal factors are significantly associated with treatment outcomes in patients with AN. Interestingly, patients with lower LGI as measured by SIRI and chloride level at baseline were more likely to experience better nutritional recovery. Given the limited understanding of reliable predictors of treatment response in AN, our results provide valuable insights into the biological mechanisms underlying nutritional rehabilitation. The clinical relevance of these findings should be further validated in larger cohorts of patients with AN to better assess improvements in nutritional status. This study highlights the importance of a more in-depth analysis of basic laboratory measurements, which may serve as cost-effective and potentially useful clinical biomarkers for monitoring progress during nutritional rehabilitation in AN. Future research should focus on targeting inflammatory pathways to improve treatment outcomes in eating disorders.

## Figures and Tables

**Table 1:** The characteristics of examined group.

Variable	N	Mean	Min–Max
Age [years]	50	14.76 ± 2.67	12–27
BMI before recovery [kg/m^2^]	49	15.12 ± 1.08	12.66–17.26
BMI after recovery [kg/m^2^]	44	17.36 ± 0.93	15.02–18.90
Duration of illness [months]	47	22 ± 31.5	3–180
Hospital stays duration [days]	50	87 ± 33	15–161
		**N**	**%**
Medication intake		43	86
Menstrual status	Primary amenorrhea	9	18
	Secondary amenorrhea	40	50
	Menstruating	1	2

BMI—body mass index; N—number; Min—minimum value; Max—maximum value.

**Table 2:** The proposed blood indicators of treatment response in the examined group.

Variable	AUC	SE	Lower Limit	Upper Limit	z	*p*-Value
Sodium	0.791	0.086	0.622	0.96	3.382	0.001
Chlorides	0.82	0.066	0.69	0.95	4.822	<0.001
fT4	0.781	0.097	0.591	0.972	2.891	0.004
Monocyte count	0.785	0.072	0.643	0.927	3.933	<0.001
MCV	0.721	0.088	0.549	0.892	2.52	0.012
NLR	0.745	0.086	0.578	0.913	2.869	0.004
MLR	0.785	0.072	0.643	0.927	3.933	<0.001
SII	0.736	0.089	0.562	0.911	2.652	0.008
SIRI	0.803	0.067	0.671	0.935	4.51	<0.001

AUC—area under the curve; SE—standard error; fT4—free thyroxine; MCV—mean corpuscular volume; NLR—neutrophil to lymphocyte ratio; MLR—monocyte to lymphocyte ratio; SII—the systemic immune-inflammation index; SIRI—the systemic inflammation response index.

**Table 3:** The proposed cut-off values for indicators of treatment response in AN.

Variable	Proposed Cut-Off Point	Younden Index
Sodium	141 mmol/L	0.54
Chlorides	104.4 mmol/L	0.56
fT4	14.96 ng/L	0.49
Monocyte count	0.31	0.42
MCV	87.1 fl	0.46
NLR	1.10	0.56
MLR	0.18	0.51
SII	266.32	0.50
SIRI	0.45	0.57

fT4—free thyroxine; MCV—mean corpuscular volume; NLR—neutrophil to lymphocyte ratio; MLR—monocyte to lymphocyte ratio; SII—the systemic immune-inflammation index; SIRI—the systemic inflammation response index.

**Table 4:** Performance of blood parameters in determining improvement of nutritional status in individuals with AN according to multiple regression analysis.

	β-Coefficient	Mean Square	F	*p*
Constant term		9.57	11.35	0.002
SIRI	−0.34	4.95	5.87	0.021
Chlorides	−0.41	7.01	8.32	0.007

SIRI—the systemic inflammation response index.

## Data Availability

The data presented in this study are available on request from the corresponding author. The data are not publicly available due to being a part of an ongoing study.

## Supplementary Materials

The following supporting information can be downloaded at https://www.mdpi.com/article/10.3390/nu17142260/s1, Figure S1: Proposed immune–metabolic–hormonal biomarkers thresholds for predicting treatment response in anorexia nervosa; Figure S2: The proposed cut-off points for inflammatory indices in determining treatment response in anorexia nervosa. The blue line represents relationship between sensitivity (True Positive Rate) and 1-specificity (False Positive Rate) for different classification threshold values. The closer this line is to the upper left corner of the graph, the better the test performance; The green line represents proposed cut-off point; The red line represents random classifier—reference point.

## Abbreviations

The following abbreviations are used in this manuscript:

AN	Anorexia Nervosa
CBC	Complete blood count
RBC	Red blood cells
WBC	White blood cells
PLT	Platelets
BMI	Body mass index
TNF-α	Tumor necrosis factor-alpha
NLR	Neutrophil-to-lymphocyte ratio
MLR	Monocyte-to-lymphocyte ratio
LGI	Low-grade inflammation
PLR	Platelet-to-lymphocyte ratio
SII	Systemic immune-inflammation index
SIRI	Systemic inflammation response index
SNRIs	Selective norepinephrine and serotonin reuptake inhibitors
SSRIs	Selective serotonin reuptake inhibitors
NHR	Neutrophil-to-high-density lipoprotein cholesterol ratio
MHR	Monocyte-to-high-density lipoprotein cholesterol ratio
PHR	Platelet-to-high-density lipoprotein cholesterol ratio
LHR	Lymphocyte-to-high-density lipoprotein cholesterol ratio
THS	Thyroid stimulating hormone
fT4	free Thyroxine
NT-ptoBNP	N-terminal pro b-type natriuretic peptide
ICD	International Classification of Diseases
PCC	Pearson correlation coefficient
ROC	Receiver operating characteristic
AUC	Area under the curve
R	Responder group
NR	Non-responder group
