# Supplementary material for: The Prognostic Value of Hematological, Immune-Inflammatory, Metabolic, and Hormonal Biomarkers in the Treatment Response of Hospitalized Patients with Anorexia Nervosa
Source: Nutrients. 2025 Jul 9;17(14):2260. doi: 10.3390/nu17142260 (PMC12299521; doi:10.3390/nu17142260)
Supplement: Supplementary file 1 [file nutrients-17-02260-s001.zip › nutrients-3694297-supplementary.pdf]

nutrients

MDPI

Supplementary materials

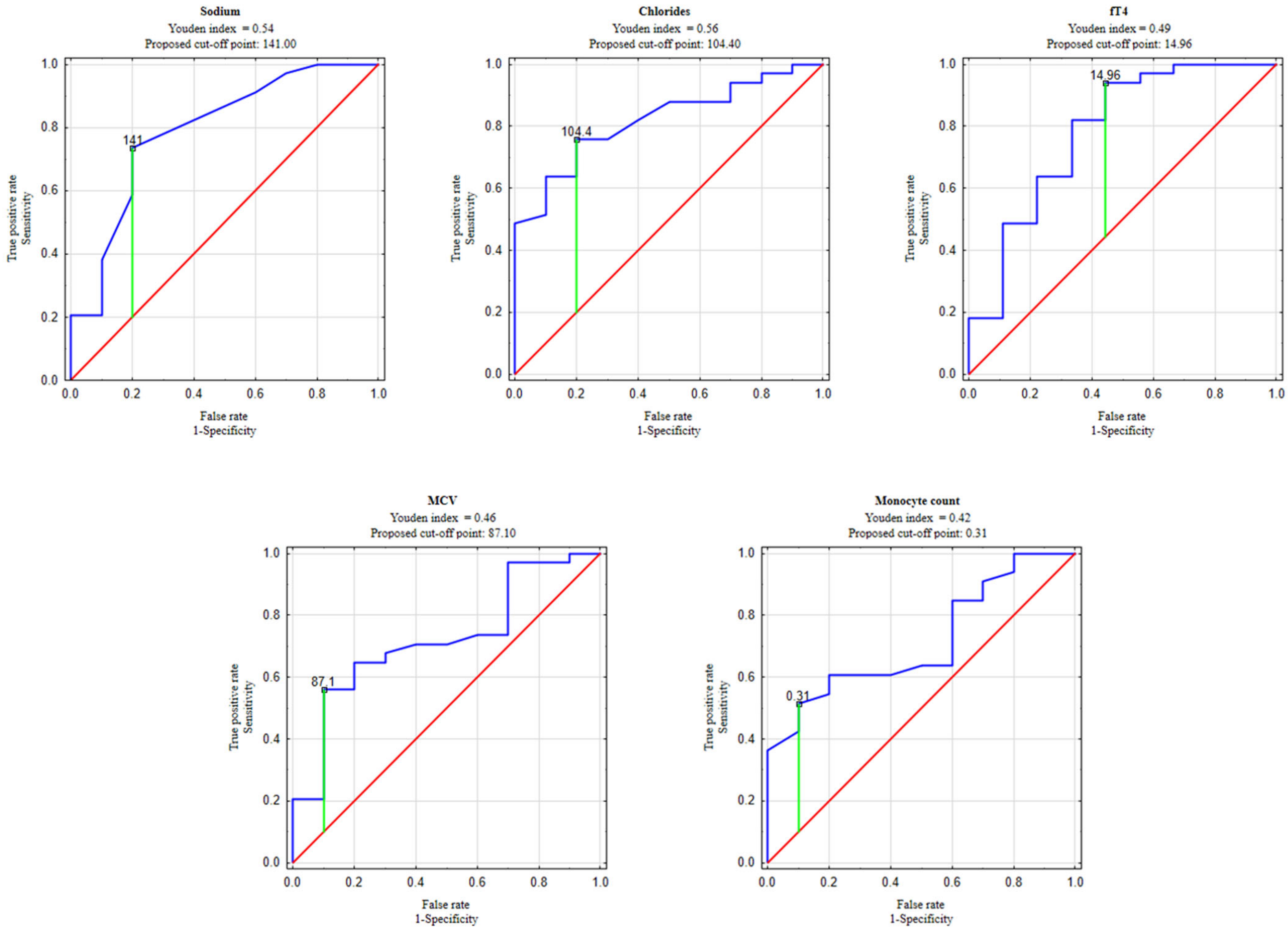

Figure S1. The proposed cut-off points for electrolytes, hormones and CBC markers in determining treatment response in anorexia nervosa

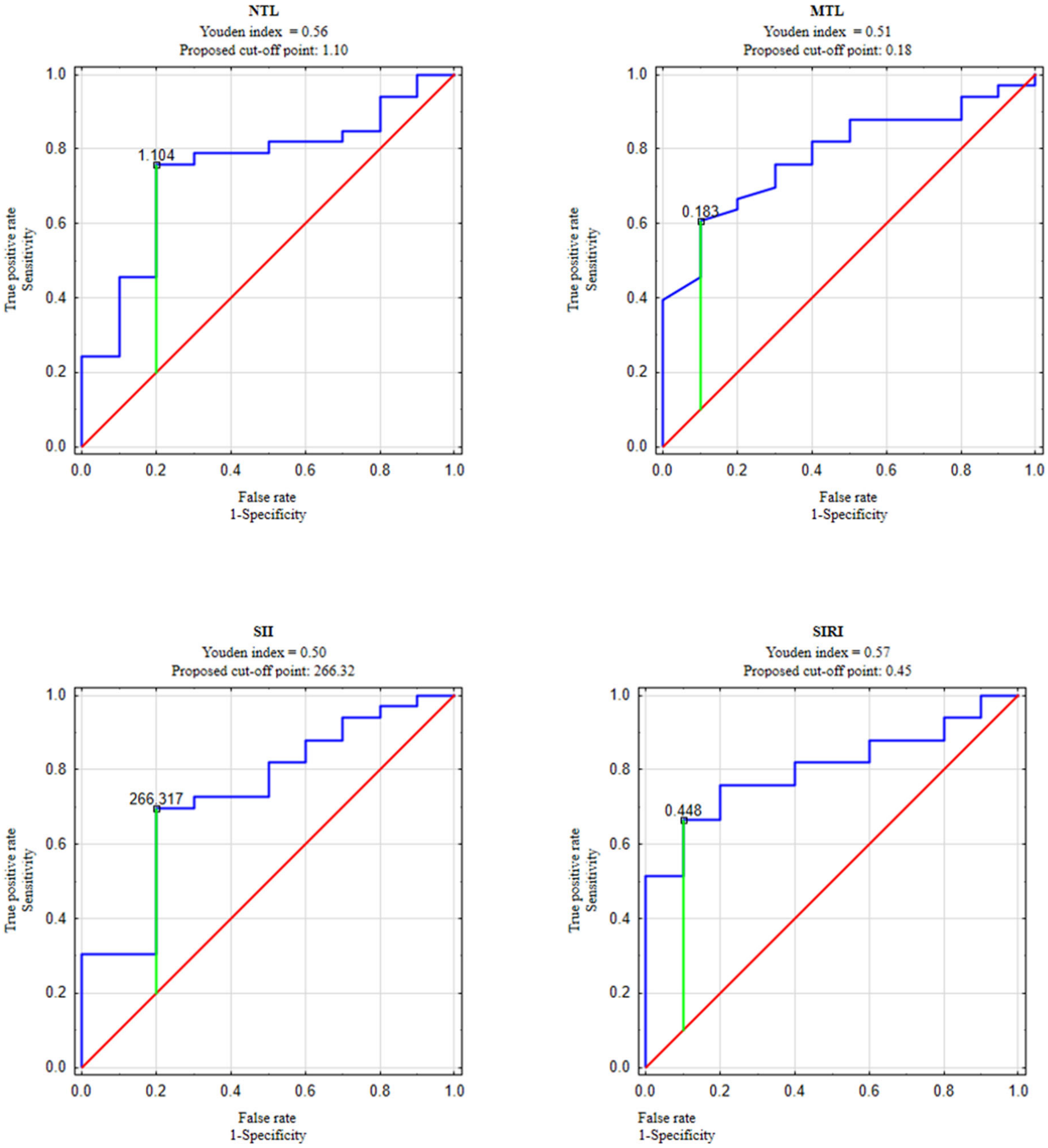

Figure S2. The proposed cut-off points for inflammatory indices in determining treatment response in anorexia nervosa
